# Apalutamide-induced severe cutaneous adverse reactions in prostate cancer: a comprehensive review of reported cases and clinical strategies

**DOI:** 10.3389/fphar.2026.1769981

**Published:** 2026-04-01

**Authors:** Taisong Wang, Shuang Liu, Renbin Yuan

**Affiliations:** 1 Southwest Medical University School of Clinical Medical Sciences, Luzhou, Sichuan, China; 2 Hospital of Chengdu University of Traditional Chinese Medicine, Chengdu, China

**Keywords:** androgen receptor antagonists, apalutamide, drug eruptions, drug therapy, prostatic neoplasms

## Abstract

**Purpose:**

Prostate cancer (PCa) is the most common fatal malignancy among men and a major cause of cancer-related death. Apalutamide, a second-generation androgen receptor inhibitor, is approved for non-metastatic castration-resistant prostate cancer (nmCRPC) and metastatic castration-sensitive prostate cancer (mCSPC). However, an increasing number of potentially life-threatening severe cutaneous adverse reactions (SCARs) associated with apalutamide have raised clinical concerns. This review aims to characterize apalutamide-induced SCARs and summarize effective management strategies based on reported cases.

**Materials and methods:**

We systematically searched PubMed, Europe PMC, and CNKI for case reports of SCARs associated with apalutamide. Keywords included “Apalutamide” and “Drug eruptions.” A total of 18 cases were identified and analyzed.

**Results:**

We reviewed the clinical characteristics and treatment of 3 cases of DRESS, 14 cases of SJS/TEN, and 1 case of AGEP, all highly suspected to be caused by apalutamide, reported globally. The most frequent SCARs associated with apalutamide are SJS/TEN. The median onset time of SCARs in these cases was 39.5 days, significantly shorter than the onset time of rash in phase III clinical trials of apalutamide. Geographically, the majority of reported cases originated from East Asia. By analyzing the treatment regimens and clinical outcomes of these patients, combined with a literature review, we proposed a set of definitive therapeutic strategies.

**Conclusion:**

Apalutamide-induced SCARs tend to occur earlier than common rashes observed in clinical trials, with a predominance of reported cases in East Asian populations. Immediate discontinuation of the suspected drug is the cornerstone of SCAR management. Supportive care, systemic corticosteroids, intravenous immunoglobulin (IVIG), cyclosporine, plasmapheresis, and TNF-α antagonists play important roles in treatment. Personalized dosing strategies based on body weight or body surface area, along with proactive rash management, may help mitigate risk and optimize therapeutic continuity.

## 1 Introduction

According to data from the World Health Organization’s International Agency for Research on Cancer (IARC) in 2022, there were 1.466 million new cases of prostate cancer worldwide, with 903,859 deaths. PCa ranks second among all male malignancies in terms of incidence (Bray et al., 2024). The American Cancer Society (ACS) estimates that in 2025, there will be 313,780 new cases of PCa in the United States, accounting for 30% of all male cancer cases. PCa has surpassed lung cancer as the most common malignancy in men (Siegel et al., 2025). The increasing incidence of PCa worldwide is significantly impacting men’s health and lives. Apalutamide is a non-steroidal androgen receptor inhibitor. It competitively binds with the androgen receptor (AR) in the context of AR overexpression and prevents AR nuclear localization as well as the binding of AR to DNA, thereby inhibiting tumor growth.

In the two phase III clinical trials (SPARTAN and TITAN), the apalutamide group showed significant improvements over the placebo group in the median metastasis-free survival (MFS) and the 2-year overall survival (OS) rate. Nevertheless, compared to the placebo group, the apalutamide group experienced a higher incidence of rash (23.8% vs. 5.5% in the SPARTAN trial; 27.1% vs. 8.5% in the TITAN trial), with the majority of reported rashes being grade 1 or 2 (Smith et al., 2018; Chi et al., 2019). It is noteworthy that subgroup analyzes of Japanese patients in these two studies showed a higher incidence of rash and grade 3 rash in the apalutamide group compared to the placebo group (Uemura et al., 2021; Uemura et al., 2022; Tohi et al., 2022; Uemura et al., 2020). This significant increase in the incidence of rashes contrasts with other second-generation AR inhibitors. For instance, in phase III clinical trials, Enzalutamide and Darolutamide did not report a significantly elevated risk of skin adverse events, and their rash incidence was typically similar to that of the placebo group (Scher et al., 2012; Hussain et al., 2018; Fizazi et al., 2019; Smith et al., 2022). This difference helps to clarify that the rashes observed clinically are not a class effect of these targeted drugs, but rather more likely to be a unique adverse reaction characteristic of apalutamide. Although no severe cutaneous adverse reactions (SCARs) were observed in the phase III clinical trial of apalutamide, in its actual application, more and more cases of severe skin adverse reactions highly suspected to be caused by apalutamide began to be reported. This fatal drug adverse event should attract further attention. Therefore, when making clinical decisions, doctors and patients can be aware that rashes are a relatively specific management focus for apalutamide.

SCARs are a type of dose-dependent hypersensitivity reaction, including Drug Reaction with Eosinophilia and Systemic Symptoms (DRESS), Stevens-Johnson Syndrome (SJS), Toxic Epidermal Necrolysis (TEN), and Acute Generalized Exanthematous Pustulosis (AGEP). All are delayed type IV hypersensitivity reactions, in which T cell receptors recognizing drug antigens presented by human leukocyte antigens, triggering the activation of distinct T cell subsets (Hung et al., 2024). Therefore, we aim to explore the clinical management strategies for SCARs caused by apalutamide in patients with PCa.

## 2 Materials and methods

**FIGURE 1:**
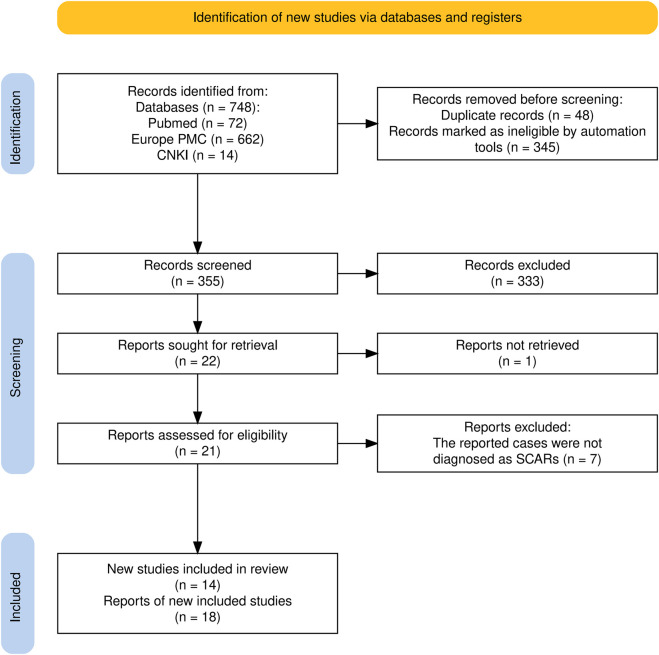
PRISMA flow diagram of the literature search and case selection process for apalutamide-associated severe cutaneous adverse reactions.

We conducted a comprehensive literature search to identify all reported cases of severe cutaneous adverse reactions (SCARs) associated with apalutamide (Figure 1). According to the World Allergy Organizations (WAO) definition of 2014, SCAR includes the following: SJS, SJS/TEN, TEN, and DRESS. The following electronic databases were systematically searched from inception to 31 December 2024: PubMed, Europe PMC, and China National Knowledge Infrastructure (CNKI). The following keywords were used: “Apalutamide,” “Drug Eruptions,” “Severe Cutaneous Adverse Reactions,” “SJS,” “SJS/TEN,” “TEN,” and “DRESS.” Boolean operators (AND, OR) were applied to refine and combine search terms. The detailed search strategy for each database is provided in Supplementary Appendix.

Studies were considered eligible for inclusion if they met the following criteria: (1) reported original case reports or case series of patients diagnosed with or highly suspected of SCARs (DRESS, SJS, TEN, SJS/TEN overlap, or AGEP) according to established diagnostic criteria; (2) apalutamide was considered the primary suspected culprit; (3) sufficient clinical data were provided to confirm the diagnosis and assess causality; (4) published in any language. Given that SCAR case reports are frequently published as letters or short communications, such publications were included provided they contained extractable patient-level data. The following publication types were excluded: editorials, conference abstracts, poster presentations, unpublished studies, review articles without original case data, and duplicate publications. In cases of overlapping patient cohorts, only the most comprehensive report was included.

All retrieved records were imported into EndNote X20 (Clarivate Analytics) for duplicate removal and subsequent screening. Two independent reviewers (Dr.Wang and Dr. Liu) performed title and abstract screening against the predefined eligibility criteria. Full texts of potentially relevant articles were then retrieved and independently assessed for final inclusion. Any discrepancies between the two reviewers were resolved through discussion; if consensus could not be reached, a third senior reviewer (Dr.Yuan) was consulted for final adjudication.

Data extraction was performed independently by two reviewers (Dr.Wang and Dr. Liu) using a standardized data collection form. The following information was extracted from each included study: (1) patient demographics (age, ethnicity, body weight, BMI); (2) Apalutamide treatment details (dose, duration, time to onset); (3) clinical features of SCARs (type of reaction, skin involvement extent, mucosal involvement, systemic symptoms); (4) Histopathological findings; (5) causality assessment tools used (ALDEN, Naranjo scale); (6) disease severity scores (SCORTEN, CTCAE grade); (7) treatment interventions (drug discontinuation, supportive care, systemic corticosteroids, IVIG, cyclosporine, plasmapheresis, TNF-α antagonists); and (8) clinical outcomes. Given the heterogeneity of included case reports and the lack of some data, a narrative synthesis approach was adopted.

In the end, a total of 18 cases of SCARs—comprising 3 cases of DRESS, 14 cases of SJS/TEN, and 1 case of AGEP—were identified and included in this review. To our best knowledge, 18 cases of SCARs associated with apalutamide have been reported worldwide, with 7 cases from Japan, nine from China, one from France, and one from Ireland. It is noteworthy that compared to other populations, Asian patients seem to be more susceptible to developing SCARs following the use of apalutamide.

## 3 The 3 cases of DRESS

In this cohort, we identified a total of 3 cases of DRESS potentially attributable to apalutamide therapy (Ducharme et al., 2022; Hsu et al., 2023). DRESS, standing for Drug Reaction with Eosinophilia and Systemic Symptoms, also known as drug hypersensitivity syndrome, is a rare and potentially life-threatening severe adverse drug reaction. Its clinical features include rash, hematological abnormalities (eosinophilia, atypical lymphocytosis), and involvement of lymph nodes and visceral organs. Clinical manifestations typically appear 2–8 weeks after medication use, and symptoms can persist for several weeks even after discontinuation of the drug. This prolonged duration may be related to T-cell mediated delayed hypersensitivity reactions (Owen and Jones, 2021; Wei et al., 2023).

In cases 1, 2, and 3, DRESS was definitively diagnosed, with apalutamide identified as the sole suspect drug causing DRESS (Table 1). The dose of apalutamide was consistent with that in the phase III trials, both being 240 mg. But the onset of rash was notably earlier (the median time to rash onset was 82 days in SPARTAN and 81 days in TITAN). The rash characteristics in the three DRESS cases were relatively typical. Rashes appeared 4–6 weeks after medication use, with widespread erythematous rashes covering over 50% of the body surface area, often accompanied by fever, facial edema, and mucosal involvement. Besides the rash, laboratory tests in all three DRESS cases showed elevated eosinophils and liver transaminases, indicating involvement of at least the hematological system and liver, with acute kidney injury also observed in the third case. It is important to note that the pathology results of DRESS skin biopsies can vary. The latter two cases were diagnosed as DRESS with suspected SJS due to the rare occurrence of extensive skin peeling and the lack of typical pathological features and target lesions. In terms of treatment, apalutamide was discontinued shortly after the onset of the rash in the first two cases, and the rashes were resolved on the 10th and 30th days, respectively, after systemic steroid therapy (Table 1).

**TABLE 1:** Cases of DRESS due to apalutamide.

Case	Case 1 (Ducharme et al., 2022)	Case 2 (Hsu et al., 2023)	Case 3 (Hsu et al., 2023)
Population	Caucasian (French)	Asian (Chinese)	Asian (Chinese)
Age	85	85	77
Onset (day)	39	28	38
Diagnosis	DRESS	DRESS with possible SJS	DRESS with possible SJS
Clinical feature	Fever, confluent erythematous plaques involving 90% of BSA, facial edema, without epidermal detachment or mucosal involvement	Fever, generalized erythematous maculopapular rash, facial edema, and periorbital sparing. Painful erythroderma and large flaccid blisters with a positive Nikolsky sign subsequently developed	Widespread flaccid bullae and denuded erosions developed on an erythematous base across the face, trunk, and limbs. The lesions were accompanied by significant skin tenderness, a positive Nikolsky sign, and mucosal erosions
Histopathology	Apoptotic keratinocytes and dermal infiltrate of eosinophils	Severe interface dermatit with numerous apoptotic keratinocytes, whole layer epidermal necrosis, and dermal epidermal separation	Severe interface dermatitis with confluent apoptotic keratinocytes, whole layer epidermal necrosis,and fermal-epidermal separation.Perivascular lymphocytes and eosinophil infiltration observed in the upper dermis
Treatment	Apa discontinued, systemic steroids	Apa discontinued, systemic steroids	NA
Outcome	Healed	Healed	NA

Apa, Apalutamide; NA, not available; BSA, body surface area.

In the treatment strategy for DRESS, promptly discontinuing all potential causative drugs and providing symptomatic supportive care are key initial steps. Systemic corticosteroids therapy is widely accepted as the standard treatment for DRESS. Upon initiation of systemic corticosteroid therapy, the recommended minimum dose is 1.0 mg/kg/day of prednisone or equivalent. Typically, symptoms such as rash, fever, and liver function impairment resolve rapidly following the initiation of systemic corticosteroid therapy. In patients who show no improvement or worsening of symptoms with oral corticosteroids, or those with significant visceral involvement, treatment with intravenous methylprednisolone may be considered. A course of pulsed methylprednisolone at a dose of 30 mg/kg/day administered intravenously for 3 days can be employed. Remarkably, even after relief of symptoms, the corticosteroids dose should be gradually tapered off, usually over a period of 6–8 weeks, to prevent relapse (Wei et al., 2023). However, retrospective studies have shown that patients receiving systemic corticosteroids therapy have higher rates of infection and sepsis, and are more likely to require intensive care compared to those receiving topical corticosteroids. Therefore, topical corticosteroids are recommended for mild cases of DRESS, while systemic corticosteroids should be reserved for patients with severe manifestations (Funck-Brentano et al., 2015). Additionally, for patients who are unresponsive to systemic corticosteroids or have contraindications, immunosuppressants like cyclosporine, intravenous immunoglobulin (IVIG), and plasmapheresis may be effective. However, due to the current lack of sufficient clinical evidence, their routine use is not recommended (Nguyen et al., 2020; Scheuerman et al., 2001; Wei et al., 2024).

## 4 The 14 cases of SJS/TEN

Through rigorous case adjudication, we confirmed a total of 14 cases of SJS/TEN potentially associated with apalutamide use (Endo et al., 2020; Sagawa et al., 2020; Huang et al., 2022; Miyagawa et al., 2022; Oda et al., 2022; Osawa et al., 2022; Wu et al., 2023; Flynn et al., 2023; Yana et al., 2023; Wang et al., 2024). Stevens-Johnson Syndrome (SJS) and Toxic Epidermal Necrolysis (TEN) are considered the most severe types of drug hypersensitivity reactions, with a mortality rate as high as 50%. Recent studies have shown that the pathogenesis of SJS/TEN is associated with cytotoxic T lymphocytes (CTL), which recognize the “culprit drug” presented by class I HLA molecules on keratinocytes, leading to severe hypersensitivity reactions through the secretion of various cytotoxic mediators. In fact, SJS and TEN are part of the same disease spectrum and are differentiated based on the affected skin area (including all blisters, partially or fully detached skin, and areas positive for Nikolsky’s sign): SJS is defined when <10% of body surface area (BSA) is involved; SJS/TEN overlap is defined when 10%–30% of BSA is involved; TEN is defined when >30% of BSA is involved. To our knowledge, there have been 14 cases of SJS/TEN associated with apalutamide reported, with 13 cases diagnosed as SJS/TEN from Japan or China, and 1 case from Ireland diagnosed as SJS/TEN overlap (Table 2). In these 18 cases of SJS/TEN, although patients had used multiple drugs prior to the onset of SJS/TEN, after evaluation using the Algorithm of Drug Causality for Epidermal Necrolysis (ALDEN) or the Naranjo Adverse Drug Reaction Probability Scale (NADRPS), and clinical speculation, apalutamide was ultimately considered the most likely cause of SJS/TEN (Table 2).

**TABLE 2:** Cases of SJS/TEN due to apalutamide.

Case	Case 1 (Ducharme et al., 2022)	Case 2 (Hsu et al., 2023)	Case 3 (Hsu et al., 2023)
Population	Caucasian (French)	Asian (Chinese)	Asian (Chinese)
Age	85	85	77
Onset (day)	39	28	38
Diagnosis	DRESS	DRESS with possible SJS	DRESS with possible SJS
Clinical feature	Fever, confluent erythematous plaques involving 90% of BSA, facial edema, without epidermal detachment or mucosal involvement	Fever, generalized erythematous maculopapular rash, facial edema, and periorbital sparing. Painful erythroderma and large flaccid blisters with a positive Nikolsky sign subsequently developed	Widespread flaccid bullae and denuded erosions developed on an erythematous base across the face, trunk, and limbs. The lesions were accompanied by significant skin tenderness, a positive Nikolsky sign, and mucosal erosions
Histopathology	Apoptotic keratinocytes and dermal infiltrate of eosinophils	Severe interface dermatit with numerous apoptotic keratinocytes, whole layer epidermal necrosis, and dermal epidermal separation	Severe interface dermatitis with confluent apoptotic keratinocytes, whole layer epidermal necrosis,and fermal-epidermal separation.Perivascular lymphocytes and eosinophil infiltration observed in the upper dermis
Treatment	Apa discontinued, systemic steroids	Apa discontinued, systemic steroids	NA
Outcome	Healed	Healed	NA

Case	Case 7 (Miyagawa et al., 2022)	Csae 8 (Oda et al., 2022)	Case 9 (Oda et al., 2022)
Population	Asian (Japanese)	Asian (Japanese)	Asian (Japanese)
Age	86	91	85
Dose (mg/d)	240	240	240
Onset (day)	28	75	50
Diagnosis	TEN	TEN	TEN
Clinical manifestation	Fever and erythema on the extremities and trunk progressed to epidermal detachment affecting more than 30% of BSA. Hemorrhagic crusts and erosions developed on the lips, with coalescence of blisters and erosions	Fever and a rash on the extremities and trunk progressed to epidermal detachment and denudation affecting more than 30% of BSA, accompanied by hemorrhagic crusts and erosions on the lips	Fever and erythema with flat atypical target lesions involved approximately 40% of BSA, with associated flaccid blisters and erosions. Oral hemorrhagic erosions and a positive Nikolsky sign were also present
SCORTEN/Naranjo	SCORTEN 2/NA	SCORTEN 2/NA	NA
Histopathology	Subepidermal blister with necrosis of individual epidermal cells	Interface dermatitis with necrosis of individual epidermal cells	Basal layer liquefactive degeneration, with subepidermal blister formation and skin peeling in some areas
Treatment	Apa discontinued, systemic PSL MPPT, IVIG, plasmapheresis	Apa discontinued Systemic PSL MPPT, IVIG.	Apa discontinued, oral PSL Antimicrobial Plasmapheresis
Outcome	Healed	Death^d^	Death^e^

Case	Case 10 (Osawa et al., 2022)	Case 11 (Wu et al., 2023)	Case 12 (Flynn et al., 2023)
Population	Asian (Japanese)	Asian (Chinese)	Caucasian (Irish)
Age	85	77	70
Dose (mg/d)	240	240	240
Onset (day)	43	14	45
Diagnosis	TEN	TEN	TEN
Clinical manifestation	Fever and erythema with flat atypical target lesions involved approximately 40% of BSA, with associated flaccid bullae and erosions. Oral hemorrhagic erosions and a positive Nikolsky sign were also present	Fever and diffuse purpuric erythema with extensive body-wide erosions were observed, along with erosions and hemorrhagic crusts on the lips and oral mucosa	Fever, pruritus, and diffuse erythema on the face, extremities, and trunk were observed, with slack bullae and epidermal detachment involving more than 30% of BSA on the neck, trunk, and limbs. Mucosal erosions were present on the eyes, lips, and scrotum
SCORTEN/Naranjo	SCORTEN 4/NA	SCORTEN 5/NA	SCORTEN 3/NA
Histopathology	Full-thickness epidermal keratinocyte necrosis with associated subepidermal blistering	NA.	Interface change, basal vacuolation, and prominent dyskeratotic keratinocytes scattered throughout the epidermis
Treatment	Apa discontinued MPPT, IVIG, plasmapheresis Ciclosporin Etanercept	Apa discontinued, systemic mPSL Antimicrobial IVIG.	Apa discontinued Systemic steroids, paraffin gel IVIG, antimicrobial
Outcome	Healed	Healed	Death^f^

Case	Case 13 (Yana et al., 2023)	Case 14 (Wang et al., 2024)	Case 15 (Wang et al., 2024)
Population	Asian (Chinese)	Asian (Chinese)	Asian (Chinese)
Age	70	77	69
Dose (mg/d)	240	240	240
Onset (day)	30	40	90
Diagnosis	TEN	SJS	SJS
Clinical manifestation	Pruritus and burning pain were associated with a widespread purplish maculopapular rash. Erosions with exudation and crusts involved the periorbital, perioral, and perianal areas, with scattered vesicular rashes on the shoulders, back, and both lower limbs	Fever and maculopapular erythema on the trunk and limbs were observed, with hemorrhagic crusts and erosions on the bilateral buccal mucosa and lips. Estimated BSA involvement was 26%	Pruritus was accompanied by maculopapular erythema that gradually spread to the entire body
SCORTEN/Naranjo	NA	NA/Naranjo 6	NA/Naranjo 6
Histopathology	NA	Interface dermatitis with whole-layer epidermal necrosis and dermal-epidermal fissure	Epidermal necrosis and perivascular infiltrated lymphocytes and eosinophils in the upper dermis
Treatment	Apa discontinued, oral antihistamines	​	​
Antimicrobial, IVIG, topical rhGM-CSF hydrogel	Apa discontinued, systemic mPSL	​	​
Adalimumab	Apa discontinued, systemic mPSL	​	​
Oral thalidomide	​	​	​
Outcome	Healed	Healed	Healed

Case	Case 16 (Wang et al., 2024)	Case 17 (Wang et al., 2024)
Population	Asian (Chinese)	Asian (Chinese)
Age	70	74
Dose (mg/d)	240	240
Onset (day)	45	2
Diagnosis	SJS	TEN
Clinical manifestation	Pruritus, maculopapular erythema, excoriations, and lichenification were observed, involving approximately 66% of BSA	Shortly after apalutamide initiation, nausea and late-onset truncal erythema occurred, which improved after drug withdrawal. Upon re-challenge, rapid progression to TEN involving >60% BSA, with necrosis and exudation of the buccal and urethral mucosa
SCORTEN/Naranjo	NA/Naranjo 6	SCORTEN 5/Naranjo 9
Histopathology	NA	NA
Treatment	Apa discontinued Systemic mPSL	Apa discontinued MPPT Adalimumab
Outcome	Healed	Death^g^

SCORTEN, severity-of-illness score for toxic epidermal necrolysis; BSA, body surface area; PSL, prednisolone; mPSL, methylprednisolone; MPPT, methylprednisolone pulse treatment; NA, not available.

Death^a^: After receiving systemic steroid treatment, IVIG, and plasmapheresis, the patient’s rash eventually improved, but the patient died from bacterial pneumonia 2 weeks later.

Death^b^: Skin detachment rapidly progressed, and the patient died of multiple organ failure 53 days after the onset of the rash.

Death^c^: The maculopapular rash and skin exfoliation stopped progressing. Due to the patient’s family refusal to further treatment, patient died of multiple organs failure.

Death^d^: The skin symptoms gradually improved,but he developed pneumocystis pneumonia and he died on day 45 of hospitalization.

Death^e^: The patient’s skin symptoms improved slowly, but due to uncontrollable infection and tuberculosis recurrence, the patient eventually died of circulatory and respiratory failure 120 days after hospitalization.

Death^f^: After treatment, the patient’s skin condition gradually improved, but the overall physical condition remained poor, unfortunately passed away following a fall resulting in hip fracture 4 weeks post discharge.

Death^g^: Owing to the rapid improvement of the lesions, the urologist advised him to recommence the apalutamide treatment for his prostate cancer, unfortunately,his cutaneous lesions reoccurred and quickly developed to TEN, he passed away due to multi-organ failure within 3 days.

In the reports of the 13 SJS/TEN cases in Asian patients, almost all patients were aged 70 and above (Table 2). A previous retrospective study from South Korea showed that patients over the age of 70 have a higher risk of developing SJS and TEN compared to other age groups, which is consistent with our findings (Yang et al., 2016). The majority of these patients were on a standard dose of 240 mg/day of apalutamide, showing no significant variance. The median time to onset of cutaneous adverse reactions in the 14 SJS/TEN cases was 41 days, notably shorter than the median onset time in the two phase III trials (Smith et al., 2018; Chi et al., 2019). Therefore, we speculate that earlier onset of skin events after taking apalutamide may indicate a poorer prognosis. In most of these cases, the majority of SJS/TEN patients were scored using the Severity-of-Illness Score for Toxic Epidermal Necrolysis (SCORTEN), with a mortality rate of 66% (4/6) in patients scoring 4 or above, which is very close to the mortality rate estimated by the SCORTEN scoring system.

The clinical features of SJS/TEN include widespread erythematous rashes and blisters starting on the face, trunk, and upper limbs, along with flat, atypical target lesions that gradually spread to the entire body. Mucosal involvement, which often occurs before skin lesions, is almost always present. The lips may show red edges, and the oral cavity may have bleeding erosions covered with gray-white pseudomembranes and crusts. Additionally, fever, conjunctival damage, pain or itching at the site of skin and mucosal involvement are common symptoms. Pathologically, the skin is characterized by subepidermal blisters, keratinocyte apoptosis, full-thickness epidermal necrosis, and minimal inflammatory infiltrate (Owen and Jones, 2021; Duong et al., 2017).

### 4.1 Supportive care

If a PCa patient exhibits suspected features of SJS/TEN after using apalutamide, immediate discontinuation of all suspect drugs, including apalutamide, is recommended as the critical first step in management. Simultaneously, early transfer to a specialized unit—such as a burn center, dermatology ward, or intensive care unit (ICU)—is recommended to facilitate optimal wound care and comprehensive supportive management (Palmieri et al., 2002). Especially during the acute phase, it is advised to avoid placement of cannulas/peripheral lines in affected skin areas. The use of silver-releasing non-adhesive wraps and air-fluidized beds, which can reduce the risk of infection, is recommended. When there is extensive skin detachment, it is also important to focus on fluid replacement, nutritional support, maintaining environmental temperature, and pain management (Lerch et al., 2018; Xia et al., 2016).

### 4.2 Systemic immunomodulating therapies

In the 14 cases of SJS/TEN caused by apalutamide, highly suspiciously, almost all patients were treated with corticosteroids or IVIG (Table 2). The primary risks associated with the use of systemic corticosteroids are sepsis following infection and inhibition of skin regrowth. However, a number of studies have shown that steroid pulse therapy in the early stages does not significantly affect the incidence of sepsis and skin regrowth, and may even reduce mortality rates (Kardaun and Jonkman, 2007; Ye et al., 2022). Additionally, corticosteroids have been effective in improving ocular complications (Palmieri et al., 2002). Yet, there have always been studies suggesting that corticosteroids do not effectively improve mortality rates (Duong et al., 2017; Jacobsen et al., 2022; Zimmermann et al., 2017). Therefore, the efficacy of corticosteroids in treating SJS/TEN still needs further exploration. In countries with limited healthcare budgets, they can be considered as a low-cost treatment option (Palmieri et al., 2002).

Similar to systemic steroid therapy, retrospective analyzes and prospective studies on the treatment of SJS/TEN with IVIG have also not found evidence of improved mortality rates. Compared to supportive care alone, IVIG showed no significant difference in terms of complications and mortality rates (Jacobsen et al., 2022; Huang et al., 2012; Schmidt et al., 2023). A meta-analysis indicated that, considering all factors, IVIG seems to be the least ideal treatment option.

Etanercept was administered in case 10 (Table 2). Despite systemic steroid therapy, IVIG, cyclosporine, and two rounds of plasmapheresis, the patient’s symptoms persisted recurrently. However, after the subsequent application of six doses of etanercept, all symptoms disappeared. Etanercept, a TNF-α antagonist, is considered an effective treatment for SJS/TEN due to its antagonism of the pro-inflammatory cytokine TNF-α (Krajewski et al., 2022). Wang et al. conducted a randomized controlled trial involving 96 SJS/TEN patients, finding that etanercept, compared to corticosteroids, successfully reduced predicted mortality rates, shortened skin healing time, and also had a lower incidence of gastrointestinal bleeding (Wang et al., 2018). In fact, in recent years, there have been increasing cases of successful treatment of SJS/TEN with etanercept (Wang et al., 2018; Wang et al., 2019; Jacobsen et al., 2022; Paradisi et al., 2014). Including case 10, most patients responded quickly to subcutaneous injections of etanercept, with skin detachment stopping within days and completely resolving within 20 days (Wang et al., 2019; Paradisi et al., 2014). Similarly, adalimumab, as a member of the TNF-α antagonists, was successfully used in case 14 to treat the rash (Yang et al., 2016). Additionally, although cyclosporine did not demonstrate satisfactory efficacy in case 10, it is still considered a drug with therapeutic potential due to its ability to inhibit the activation of CD8 T cells (Table 2). Cyclosporine has been found to improve the mortality rate of SJS/TEN in some retrospective studies. However, more randomized controlled trials are needed to further support this conclusion (Jacobsen et al., 2022; Lee et al., 2017; Shah et al., 2021).

### 4.3 Combination therapies

Regarding the combination therapy for SJS/TEN, corticosteroids combined with IVIG is currently the most widely used regimen. Despite ongoing debates about the efficacy of corticosteroids and IVIG alone, significant survival benefits have been observed when both are used together (Yang et al., 2022; Yamane et al., 2016). Tian et al. revealed through retrospective studies that adding etanercept to the routine treatment of systemic corticosteroids combined with IVIG further shortened the disease recovery time, reduced the total dose of corticosteroids, and showed no significant adverse reactions during 6 months of follow-up. This provides a promising option for SJS/TEN patients (Tian et al., 2022).

### 4.4 Plasmapheresis therapy

Aside from case 10, patients in cases 4, 7, and 9 experienced a certain degree of alleviation in skin symptoms following plasmapheresis (Table 2). In fact, plasmapheresis can remove drugs, autoantibodies, immune complexes, and the cytotoxic mediator Fas ligand (FasL) from the plasma, thereby inhibiting the apoptosis of keratinocytes mediated by Fas-FasL interaction and, in principle, slowing the progression of SJS/TEN. This has been confirmed in some past cases (Oda et al., 2022; Han et al., 2017). In a prospective observational study involving 28 SJS/TEN patients, plasmapheresis showed advantages in improving mortality rates and shortening ICU stay compared to corticosteroids and IVIG (Rathkopf et al., 2013). Indeed, in cases 4, 7, 9, and 10, the patients’ skin lesions improved significantly after several rounds of plasmapheresis. Notably, in case 9, Hidemasa et al. measured the plasma concentration of apalutamide before and after plasmapheresis and unexpectedly found that the plasma concentration of apalutamide increased after the plasmapheresis procedure (Table 2). They analyzed that the distribution of drugs in the body might be affected by factors such as dosage, intrinsic clearance rate, and nutritional status. After being absorbed into the body, apalutamide distributes to the extravascular space. Following plasmapheresis, the apalutamide that had distributed outside the blood vessels re-enters the bloodstream, resulting in an increase in its plasma concentration. The improvement in the patient’s skin symptoms might be related to the removal of FasL through plasmapheresis (Oda et al., 2022). We observed in the aforementioned cases that the skin symptoms continued to progress initially after discontinuing apalutamide, which might be due to its long half-life. Phase I trials have shown that the half-life of apalutamide is approximately 86.8 h after a single dose of 240 mg. Therefore, early initiation of plasmapheresis might help in timely halting the progression of the disease (Rathkopf et al., 2013).

## 5 The 1 cases of AGEP

To our knowledge, only one confirmed case of apalutamide-induced AGEP has been documented in the literature to date (Honda et al., 2022). AGEP is a rare, acute, and severe cutaneous adverse reaction, intrinsically a delayed hypersensitivity reaction mediated by T cells to specific drugs or other triggers. It typically has a sudden onset, with skin symptoms rapidly subsiding within a few days after discontinuation of the offending drug. The clinical characteristics of AGEP include high fever, rapid onset of edematous erythema and pustular rash, increased neutrophils, without involvement of internal organs, generally resulting in a good overall prognosis.

Case 18 is the first reported case of AGEP caused by apalutamide (Table 3). The patient’s clinical presentation and histopathology were typical, allowing for a diagnosis based on these characteristics. Apalutamide, as the only “suspect” drug, was discontinued on the third day after the onset of the rash. The rash improved after 14 days of systemic steroid treatment (Table 3).

**TABLE 3:** A case of AGEP due to apalutamide.

Case	Case 18 (Honda et al., 2022)
Population	Asian (Japanese)
Age	72
Dose (mg/d)	240
Onset (day)	41
CTCAE grade	3
Clinical manifestation	Fever, hypotension, and generalized edema were observed, with edematous erythema involving the trunk, forearms, and legs (covering >30% BSA). Multiple small pustules developed within the erythematous areas
Histopathology	Subcorneal and intraepidermal pustules admixed with many eosinophils
Treatment	Apa discontinued, systemic mPSL, MPPT
Outcome	Healed

CTCAE: common terminology criteria for adverse events, version 5.0.

Since AGEP is typically self-limiting, its main treatment involves the immediate cessation of the suspected drug. Additionally, some topical corticosteroids, antihistamines, and supportive care may be administered. For severe cases, systemic steroid therapy is also recommended. A recent retrospective study showed that for severe AGEP patients with contraindications to steroid use, cyclosporine is not inferior to corticosteroids in controlling rash progression and accelerating disease recovery (Yanes et al., 2020). Furthermore, TNF-α antagonists such as Secukinumab and Infliximab have also successfully treated refractory cases that were unresponsive to drug discontinuation, steroid therapy or supportive care, demonstrating the potential of biologic agents in the treatment of AGEP (Zhang et al., 2021; Meiss et al., 2007).

## 6 Discussion

Firstly, SCARs represent a spectrum of delayed, T-cell-mediated hypersensitivity reactions that, although rare, carry substantial morbidity and mortality. These cases reviewed herein—encompassing DRESS, SJS/TEN, and AGEP—highlight the diverse clinical manifestations and severities within this spectrum. Understanding that these are distinct immunopathological entities is paramount for prompt diagnosis and intervention, as the window for effective treatment is often narrow.

Among the 18 reported cases of SCARs, a total of seven patients ultimately died, all of whom were within the SJS/TEN spectrum. Of these, 2 deaths were directly attributable to the progression of SCARs itself, characterized by rapid skin detachment and subsequent multiorgan failure, including one case of fatal re-challenge after premature resumption of apalutamide. The remaining 5 deaths were indirectly related, primarily occurring after initial skin improvement but complicated by secondary infections (such as bacterial pneumonia and pneumocystis pneumonia, n = 3), treatment withdrawal (n = 1), or other indirect factors (n = 1). This highlights that while aggressive management of cutaneous symptoms is crucial, vigilance for and prevention of secondary complications, particularly infections, are equally important determinants of patient survival.

### 6.1 Proposed mechanism of apalutamide-induced SCARs

Of note, in all SCAR case reports that documented prior medication history, no new drugs had been introduced within the 6 months preceding apalutamide initiation. Based on a comprehensive review of patients’ medication histories, apalutamide was implicated as the sole suspected causative agent in nearly all instances. Furthermore, no conclusive evidence supported a contributory role of drug–drug interactions in exacerbating the severity of these cutaneous adverse reactions. To date, the specific mechanism by which apalutamide causes rashes remains unclear. However, experimental results from Ji et al. using a mouse drug allergy model support the hypothesis that the 2-cyanopyridine moiety of apalutamide may form a hapten with cysteine residues in proteins, leading to a delayed hypersensitivity reaction and consequently causing rashes (Ji et al., 2020). Similarly, SCARs are also related to delayed hypersensitivity reactions that are dose-dependent. Therefore, we speculate that in certain specific conditions, the 2-cyanopyridine part of apalutamide’s structure can cause rare but fatal SCARs.

### 6.2 Management after apalutamide-induced SCARs

For patients diagnosed with SCARs—particularly SJS/TEN—permanent discontinuation of apalutamide is considered the safest and most strongly recommended course of action. In such severe reactions, re-exposure to the suspected drug carries an extremely high risk of mortality, necessitating a transition to alternative therapeutic regimens. The potentially fatal consequence of re-challenge is starkly illustrated by Case 17 in our series, where a patient with initial improvement following drug withdrawal experienced rapid progression to TEN upon apalutamide re-administration, ultimately succumbing to multi-organ failure within 3 days despite intensive treatment. Conversely, in Case 18 (AGEP), all medications except apalutamide were resumed after significant rash improvement. Given that the patient had high-risk mHSPC, the medical team selected abiraterone as sequential therapy, which was initiated on day 53 after apalutamide discontinuation, with no recurrence of rash thereafter.

Furthermore, the unique 2-cyanopyridine moiety in the structure of apalutamide is responsible for its covalent binding to proteins and subsequent immune activation—a feature not shared by other novel androgen receptor antagonists such as enzalutamide and darolutamide. We therefore hypothesize that this structural difference may translate into a lower risk of cross-reactivity for cutaneous toxicity. Accordingly, we propose that abiraterone, enzalutamide, and darolutamide represent potential alternative agents for patients who must discontinue apalutamide due to SCARs.

### 6.3 Dose individualization for apalutamide

A critical finding from our analysis is the potential for dose individualization based on ethnicity and body size. In the SPARTAN trial involving apalutamide, the average body weight of the 801 participants in the trial group was 87.3 kg. Among them, the 34 Japanese patients who developed rashes had an average body weight of 63.9 kg. The higher per-kilogram drug exposure in these patients may explain the increased incidence of rashes among Japanese patients treated with apalutamide (Smith et al., 2018). Currently, most reported cases of apalutamide-related SCARs involve Asian men over 70 years of age, with a more rapid onset compared to the cutaneous adverse events observed in phase III clinical trials.

Emerging real-world evidence further substantiates this hypothesis. Suzuki et al. investigated 128 Japanese patients with mCSPC and demonstrated that apalutamide dose per body weight (mg/kg) was a superior predictor of skin rash incidence and severity compared to absolute dose (Suzuki et al., 2025). Using ROC curve analysis, they identified an optimal cutoff of 3.33 mg/kg. Patients receiving doses above this threshold exhibited significantly higher rates of any-grade rash (63.6% vs. 41.9%, P = 0.021), grade ≥3 rash (19.7% vs. 6.5%, P = 0.037), and rash-related discontinuation (30.3% vs. 14.5%, P = 0.023). Notably, the incidence of grade ≥3 rash in the low-dose group (≤3.33 mg/kg) was 6.5%, comparable to the 5.2% observed in the global TITAN population. Importantly, no significant difference in progression-free survival was observed between groups, suggesting that weight-based dose reduction mitigates rash risk without compromising efficacy.

Akagi et al. further refined this approach by incorporating body surface area (BSA) into dose optimization (Akagi et al., 2025). In their cohort of 63 Japanese patients, relative dose intensity adjusted for BSA (RDI/BSA) showed slightly better predictive performance for rash than RDI/kg (AUC: 0.716 vs. 0.694). They established two critical cutoffs: age >72 years and RDI/BSA ≥56. Patients with both risk factors exhibited a rash incidence of 79.3%, compared to only 7.7% in those with neither. Crucially, among patients requiring dose reduction due to rash, total RDI/BSA decreased to 36–55 (equivalent to 120–180 mg continuous dosing), yet PSA response and PFS remained comparable to those on full-dose therapy, further validating the feasibility and safety of dose reduction.

Zhu et al. provided additional insights through therapeutic drug monitoring (TDM) (Zhu et al., 2025). In 24 patients receiving a reduced dose of 180 mg apalutamide, they observed substantial inter-individual variability in steady-state trough concentrations (apalutamide: 0.517–7.27 μg/mL; N-desmethyl apalutamide: 1.78–8.32 μg/mL). Despite receiving only 75% of the standard dose, patients achieved a median PSA reduction of 97.98% at week 4, with a notably lower rash incidence than that reported for the 240 mg dose. These findings suggest that TDM-guided individualized dosing may optimize the therapeutic index of apalutamide, particularly for patients with low body weight or distinct metabolic profiles.

Collectively, these studies support a shift from fixed-dose to personalized apalutamide dosing strategies based on body weight (mg/kg), body surface area (mg/m^2^), or therapeutic drug monitoring. Integrating the findings of Suzuki et al. (optimal dose-per-weight cutoff: 3.33 mg/kg) and Akagi et al. (rash group median BSA: 1.65 m^2^), we propose that patients with body weight <60 kg or BSA <1.65 m^2^—particularly elderly Asian men—represent a high-risk population for apalutamide-induced SCARs. For such patients, an initial reduced dose of 180 mg or 120 mg should be considered to mitigate the risk of life-threatening SCARs while preserving antitumor efficacy.

### 6.4 Limitations in apalutamide SCAR case reporting

Furthermore, our review identifies significant limitations in the quality and consistency of reported case data, which hinders a more robust retrospective analysis. The frequent absence of critical data including body weight, past history, stages of PCa, and combination drug regimens. This information could be instrumental in retrospective analyzes to further identify risk factors for SCARs caused by apalutamide. For instance, data from phase III clinical trials and some retrospective studies, indicate that lower body weight and BMI are high-risk factors for skin adverse events caused by apalutamide, although most of these 18 cases did not describe the patients’ height and weight (Sasaki et al., 2022). Additionally, the use of tools like the lymphocyte transformation test (LLT), the algorithm of drug causality for epidermal necrolysis (ALDEN), and the Naranjo scale can help in identifying the “culprit drug”. The use of SCORTEN and CTCAE grading can assist doctors in assessing the severity and prognosis of the patient’s condition.

### 6.5 Extending rash management recommendations

Several evidence-based recommendations have been published to guide clinicians in the identification and management of apalutamide-related rash (non-SCARs rashes) (Shore et al., 2025; Birtle et al., 2024). While existing recommendations provide a robust framework for managing non-severe apalutamide-related rashes, our review extends this framework to include SCARs.

First, while previous guidance primarily addresses non-severe rashes, our review specifically focuses on SCARs—a rare but life-threatening endpoint not observed in clinical trials. By systematically analyzing 18 reported SCAR cases, we provide a detailed characterization of the clinical features, histopathological findings, and treatment outcomes for DRESS, SJS/TEN, and AGEP, thereby extending the existing framework to encompass the full spectrum of apalutamide-related cutaneous toxicity.

Second, our analysis reinforces the critical importance of immediate drug discontinuation and further emphasizes that for SJS/TEN, permanent discontinuation is mandatory, as re-challenge carries an extremely high risk of mortality, as tragically illustrated by Case 17 in our series.

Third, our review provides a comprehensive synthesis of emerging evidence on personalized dosing strategies, which complements the existing rash management guidelines by addressing prevention at the level of initial prescribing. We support a risk-stratified approach to dose selection. Specifically, we propose that patients with low body weight (<60 kg) or small body surface area (<1.65 m^2^)—particularly elderly Asian men—may benefit from an initial reduced dose of 180 mg or 120 mg to mitigate SCAR risk while preserving antitumor efficacy.

Fourth, our review addresses an important gap in the literature by discussing alternative agents for patients who must permanently discontinue apalutamide due to SCARs. Based on structural differences (the 2-cyanopyridine moiety unique to apalutamide), we hypothesize that enzalutamide, darolutamide, and abiraterone may represent safer alternatives with lower risk of cross-reactivity for cutaneous toxicity.

## 7 Conclusion

In this comprehensive review, we analyzed all globally reported cases of apalutamide-induced SCARs, comprising 3 cases of DRESS, 14 cases of SJS/TEN, and 1 case of AGEP. SJS/TEN was the most frequently observed SCAR associated with apalutamide. The median time to onset of SCARs was 39.5 days, significantly shorter than the rash onset reported in phase III clinical trials of apalutamide. Geographically, the majority of documented cases originated from Japan and China, consistent with subgroup analyses of phase III trials indicating a higher incidence of rash in East Asian participants. However, this observation may be influenced by differences in pharmacovigilance practices and reporting awareness. Nevertheless, special vigilance is warranted in Asian patients who develop rapid-onset symptoms following apalutamide initiation.

Immediate discontinuation of the suspected drug remains the critical first step in the management of all SCARs. We recommend that when SCARs are suspected, comprehensive clinical information—including patient weight, body mass index, allergy history, and concurrent medications—should be promptly documented. Standardized assessment tools such as ALDEN, the Naranjo scale, SCORTEN, and CTCAE grading should be employed to objectively evaluate causality, severity, and prognosis.

For the treatment of SCARs, supportive care and systemic corticosteroids remain the mainstay of therapy. Although cyclosporine, TNF-α antagonists, and plasmapheresis have demonstrated promising results in select cases, further large-cohort prospective studies are needed to establish their efficacy and safety.

In summary, apalutamide-induced SCARs, though rare, carry substantial mortality. Early recognition, prompt drug discontinuation, and individualized risk-stratified management are essential to optimize patient outcomes.
