# Supplementary material for: Apalutamide-induced severe cutaneous adverse reactions in prostate cancer: a comprehensive review of reported cases and clinical strategies
Source: Front Pharmacol. 2026 Apr 1;17:1769981. doi: 10.3389/fphar.2026.1769981 (PMC13079589; doi:10.3389/fphar.2026.1769981)
Supplement: Supplementary file 1 [file Supplementaryfile1.docx]

The detailed search strategy for each database is as follows:

- Pubmed: ("apalutamide"[Supplementary Concept] OR "apalutamide"[All Fields]) AND ("Drug Eruptions"[Mesh] OR "Drug Hypersensitivity Syndrome"[Mesh] OR "Stevens-Johnson Syndrome"[Mesh] OR "Acute Generalized Exanthematous Pustulosis"[Mesh] OR "Severe Cutaneous Adverse Reactions"[All Fields] OR "SCAR"[All Fields] OR "DRESS Syndrome"[All Fields] OR "SJS"[All Fields] OR "TEN"[All Fields] OR "rash"[All Fields] OR "skin rash"[All Fields] OR "drug eruption"[All Fields])
- Europe PMC: ("apalutamide" OR "ARN-509") AND ("Drug Eruptions" OR "Drug Hypersensitivity Syndrome" OR "Stevens-Johnson Syndrome" OR "Acute Generalized Exanthematous Pustulosis" OR "Severe Cutaneous Adverse Reactions" OR "SCAR" OR "DRESS" OR "SJS" OR "TEN" OR "rash" OR "skin rash" OR "drug eruption")
- CNKI: (SU='阿帕他胺' OR SU='阿帕鲁胺' OR SU='阿帕他胺片') AND (SU='药物超敏反应综合征' OR SU='DRESS' OR SU='Stevens-Johnson综合征' OR SU='SJS' OR SU='中毒性表皮坏死松解症' OR SU='TEN' OR SU='急性泛发性发疹性脓疱病' OR SU='AGEP' OR SU='严重皮肤不良反应' OR SU='SCAR' OR SU='皮疹' OR SU='皮肤不良反应')
